# Elastic Abdominal Binders Reduce Cesarean Pain Postoperatively: A Randomized Controlled Pilot Trial

**Published:** 2018-05-18

**Authors:** Jamie L. Gustafson, Fanglong Dong, Jennifer Duong, Zachary C. Kuhlmann

**Affiliations:** 1 University of Kansas School of Medicine-Wichita, Department of Obstetrics and Gynecology; 2 Western University of Health Sciences, Pomona, California

**Keywords:** Cesarean section, compression bandages, abdominal wall/surgery, postoperative pain, postpartum hemorrhage

## Abstract

**Background:**

A potential non-pharmacologic way to reduce postoperative pain and bleeding is using an abdominal binder during postoperative recovery. This study aims to determine the effect an elastic abdominal binder has on postoperative pain and hemorrhage after cesarean delivery.

**Methods:**

A randomized, single-site, pilot trial was conducted at two prenatal care clinics and an academic hospital in Kansas. Beginning in April 2013, 60 patients were enrolled if delivering via cesarean. Participants were randomized to receive an abdominal binder or to a control group (did not use binder). Pain levels were reported by questionnaire one day after surgery using a 0 to 10 scale, with 10 being the worst pain. Patient characteristics and blood loss were assessed by medical record review.

**Results:**

Of the 56 patients completing the study, 29 (51.8%) were randomized to the binder group and 27 (48.2%) were randomized to the control group. The binder group reported significantly lower pain score (p = 0.019) and average pain score (p = 0.024). There was no difference in body mass index, age, previous surgery, infant birth weight, estimated blood loss, and average dose of pain medication during the first 24 hours after the cesarean delivery between the two groups. There was no difference in pre- and post-operative hemoglobin levels by treatment group (p = 0.406).

**Conclusions:**

Abdominal binders may be associated with improved postoperative pain scores but did not affect postoperative hemorrhage.

## Introduction

Postpartum hemorrhage after cesarean delivery is defined as a blood loss of greater than 1000 milliliters, a decline in hematocrit levels of 10%, or symptoms from blood loss necessitating a blood transfusion.^1^–^3^ Primary obstetrical hemorrhages occur within the first 24 hours following delivery and are estimated to occur in 1 to 6% of all deliveries.^1^,^4^,^5^ Postpartum hemorrhage continues to be the leading cause of maternal mortality worldwide and one of the top three causes of maternal death in the United States.^1^,^5^,^6^ Hemorrhage that necessitates transfusion can lead to multiple infectious and non-infectious health problems.^7^ Therefore, the avoidance of transfusion due to blood loss after surgery is ideal.

Postpartum patients delivering by cesarean are a unique subset of postoperative patients with specific risks and needs. Cesarean patients are in a unique situation in that they must care for a newborn infant immediately following surgery. Postoperative pain can affect the ability to sleep and lead to frequent nighttime awakenings, which can affect daytime functioning and maternal-infant interactions.^8^,^9^ Women with greater pain are less likely to breastfeed. Although narcotics play an important role in postoperative pain control, there are potentially serious adverse reactions to these types of medications, such as opioid-related respiratory depression,^10^ sedation, and pruritus.^11^ Long-term use of narcotics may lead to gastrointestinal dysfunction like constipation and ileus/bowel complications.^12^ In addition, postpartum patients need to ambulate early to reduce the risk of thrombosis.^10^

A potential non-pharmacologic way to reduce postoperative pain and diminish postpartum bleeding is with the use of an abdominal binder during postoperative recovery.^13^ The binder is a soft elastic band, which attaches around the abdomen and adjusts to different abdominal circumferences by overlapping and attaching with Velcro. One theory regarding pain control is that an abdominal binder provides sufficient circumferential compression to reduce stress on the wound during transfers and ambulation. Another theory is that the binder provides sensory input when in contact with the skin, and that the sensory signals override the neural pathways carrying pain signals to the brain to some extent. The hemorrhage-prevention theory is that the mild pressure from the binder will assist the uterus to remain contracted as it begins the process of involution and provide mild tamponade of blood vessels in the wound.

Few randomized controlled studies reporting postoperative outcomes compared to a control group were found regarding use of abdominal binder after cesarean delivery. Cheifetz et al.^13^ conducted a study which demonstrated the benefit of abdominal binders, in which patients who wore binders after major abdominal surgery reported unchanged pain and postoperative distress. Two randomized studies reported conflicting outcomes regarding use of abdominal binders for managing postoperative pain and blood loss after cesarean delivery. Gillier et al.^14^ found no significant difference in pain between their two study groups on postoperative day one, although a slight, non-significant difference was noted postoperative day two. In contrast, Ghana et al.^15^ found patients receiving abdominal binders reported less postoperative pain and less blood loss. Our randomized controlled trial aimed to evaluate the effect of abdominal binder use on postoperative pain and hemorrhage among patients undergoing cesarean delivery. We hypothesized that patients in the intervention group will report less postpartum pain, less volume of estimated blood loss, and less pain interfering with daily activities postpartum.

## Methods

This was a randomized controlled, single-site, pilot trial. The study was conducted at a teaching hospital in Wichita, Kansas with a goal of enrolling sixty patients over one year. The patients were recruited consecutively from two local clinics; one clinic was a private clinic and the other was a clinic staffed by resident physicians. This study was approved by two local institutional review boards and has been registered on clinicaltrials.gov as a prospective randomized controlled trial with the identification number NCT01786330.

Women receiving prenatal care at either of the two clinics and planned to deliver via cesarean were eligible to participate in the study. Additional inclusion criteria included cesarean delivery at term (at least 39 weeks gestation) scheduled in advance, singleton gestation confirmed by ultrasound in the current pregnancy, aged 18 – 39 years old, were able to read and understand spoken English, and had a body mass index of 20kg/m^2^ to 40 kg/m^2^ pre-pregnancy or at the first prenatal visit.

Exclusion criteria included bleeding disorder or use of anticoagulants, methadone usage, abnormal placenta (placenta previa or placenta accreta), preoperative hemoglobin less than 10mg/dL, or chorioamnionitis (intrauterine infection). Patients that had chronic pain syndrome, defined as participating in formal chronic pain management within the past year, were excluded from the study. Two investigators reviewed each patient’s eligibility, and obtained informed consent from the patient to participate in the study. A patient was excluded from the study if onset of labor occurred prior to the time when the cesarean was scheduled, or if the following complications developed during the cesarean: placental abnormality (placenta accreta, increta, or percreta), vasa previa, cesarean hysterectomy due to severe hemorrhage, or organ damage (cystotomy, enterotomy, ureteral injury).

After obtaining informed consent, one-to-one randomization was used to assign 30 women to the intervention and 30 women to the comparison group. A sample size of 30 per group was considered adequate for a pre-testing/feasibility study of such an intervention.^16^,^17^ In regard to pain outcomes, the study was powered adequately for a reasonable improvement in pain scores. The following estimate was drawn from published results of the instrument for measuring the primary outcome for improving pain control, the Brief Pain Inventory-Short Form, in postoperative patients.^18^–^20^ For the question about pain experienced “on average,” the studies found pain scores of approximately 4 on the instrument’s 0 – 10 scale, with a standard deviation of approximately 2. If the binder was associated with a drop in average pain score of 1.5 points on the scale, (i.e., a score of 2.5 in the intervention group versus 4.0 in the control group), there would be a statistically significant result with 30 participants per group using an alpha of .05 and power of 80%.^17^ Research assistants generated the allocation sequence and randomization size, which were concealed to clinical investigators until interventions were assigned. Investigators enrolled eligible participants into the study, and research assistants assigned subjects to their respective groups. Random numbers were generated by computer in standard fashion and assigned by the research staff using IBM SPSS, Version 20, SPSS, Inc. (Chicago, IL).

Participants assigned to the intervention group received an elastic abdominal binder immediately postoperative and were instructed to wear the binder for the first 24 hours postoperatively. The women assigned to the control group received usual postoperative care, but agreed to data collection procedures associated with the study. In addition, the control group participants’ physicians were allowed to prescribe a binder postoperatively if they believed one was indicated or upon patient request. Women in both study groups received pain medication as per the orders of their physicians.

A medical record review included the collection of the obstetrical information and reason for cesarean delivery. These variables include basic descriptive information about the pregnancy and delivery, such as the reason that the cesarean was scheduled in advance and the mother’s age. Medications and dosages administered to the patient within the 24-hour postoperative period were collected and postoperative bleeding and pain outcomes were recorded.

Each participant completed a questionnaire at 24 hours postoperatively (Appendix A). The one-page questionnaire briefly addressed bleeding and pain control. Pain was assessed using the Brief Pain Inventory-Short Form.^21^–^23^ On the questionnaire, the Brief Pain Inventory-Short Form was represented by items 3 – 8 with the exception of item 8d which was analyzed separately. Because patients normally do not perform work for pay or household work during the postoperative period, other investigators studying postoperative pain have deleted the item on pain interference with work from the Brief Pain Inventory-Short Form.^22^ Instead, investigators modified the work interference item to capture a key aspect of the work of being a new mother, feeding the baby.

Postoperative blood loss was calculated as the difference between documented pre-operative hemoglobin concentration and postoperative hemoglobin concentration (lowest documented concentration during the hospital stay). Participants also were provided a bedside log to document pad use during the first 24 hours postoperative.

The primary outcome was postoperative pain, which was assessed using the Brief Pain Inventory-Short Form. The Brief Pain Inventory-Short Form is a widely-used instrument which has good psychometric properties in assessing pain in surgical patients.^21^–^23^ The secondary outcome measure was postoperative blood loss by changes in hemoglobin concentration (difference between pre-operative hemoglobin concentration and lowest hemoglobin concentration documented postoperative). The number of pads used for vaginal bleeding and discharge during the postoperative period was tracked using a bedside log, which was completed by the participant.

### Statistical Analysis

Data were analyzed using IBM SPSS, Version 20, SPSS, Inc. (Chicago, IL). Key continuous variables included number of pads used and the Brief Pain Inventory-Short Form subscale scores for pain severity and pain interference with function. Results for the intervention group versus the control group were compared using paired t-tests if the data were normally distributed and with Mann-Whitney tests if the data were skewed. Proportions were compared using Pearson’s Chi-square or by Fisher’s Exact tests when expected values in any cell were less than five. All statistical analyses were two-sided. P-value of less than 0.05 was considered statistically significant. An intention to treat analysis was conducted. Participants were analyzed in the group to which they were randomized.

## Results

**Figure 1:**
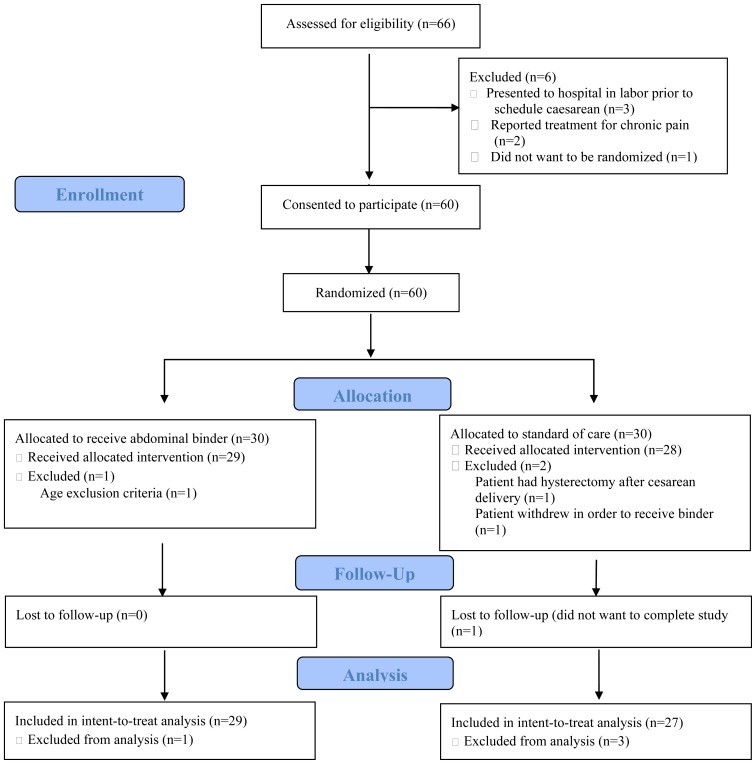
Flow diagram of participant randomization into trial.

There were 60 participants consented and randomized to either the control group or the intervention group (binder group). Of the 56 participants completing the study, 29 (51.8%) were randomized to the binder group and 27 (48.2%) were randomized to the control group. Four participants were excluded after randomization (Figure 1). Two participants crossed over to the binder group: one participant requested use of a binder and one participant was given a binder by medical staff. An intent-to-treat analysis was performed on 56 patients.

Demographics and clinical characteristics for participants completing the study are presented in Table 1. Indication for cesarean for most participants was previous cesarean (n = 50 of 56, 89.3%). Almost all participants received an epidural (n = 55, 98.2%). There was no difference in body mass index (BMI), age, previous surgery, infant birth weight, estimated blood loss, and average dose of pain medication during the first 24 hours after the cesarean delivery between the two groups. No statistically significant difference in the average dose of pain medication was found. There was also no difference in type of regional anesthesia used.

**Figure 2:**
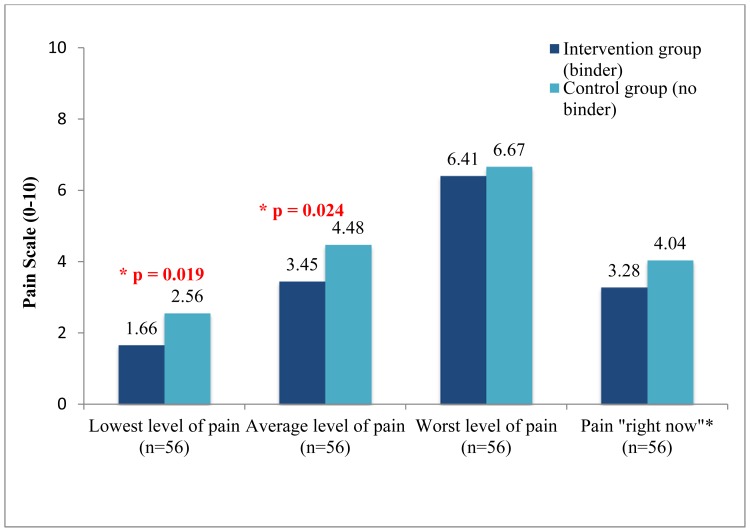
Average pain scores for lowest level of pain, average level of pain, worst level of pain, and pain at the time of assessment (24 hours postoperatively) as indicated by the control group (green) and the intervention group (blue). *Larger value represents worse self-reported pain 24 hours after Cesarean.

On average, participants in the binder group reported significantly lower pain scores than participants in the control group. The average score of participants who responded “lowest level of pain felt postoperatively” was 1.66 ± 1.47 for the binder group and 2.56 ± 1.22 for the control group (p = 0.019; Figure 2). The binder group also reported significantly lower “average pain” scores, 3.45 ± 1.74 compared to 4.48 ± 1.60 for the control group (p = 0.024, Figure 2). “Worst level of pain” and “pain right now” also were lower among women receiving the binder treatment; however, results were not statistically significant. There was not a statistically significant difference in pain interference with activities like walking (Table 2). Pain interference with feeding the baby was lower among participants receiving the binder, with results nearing statistical significance (p = 0.078).

**Figure 3:**
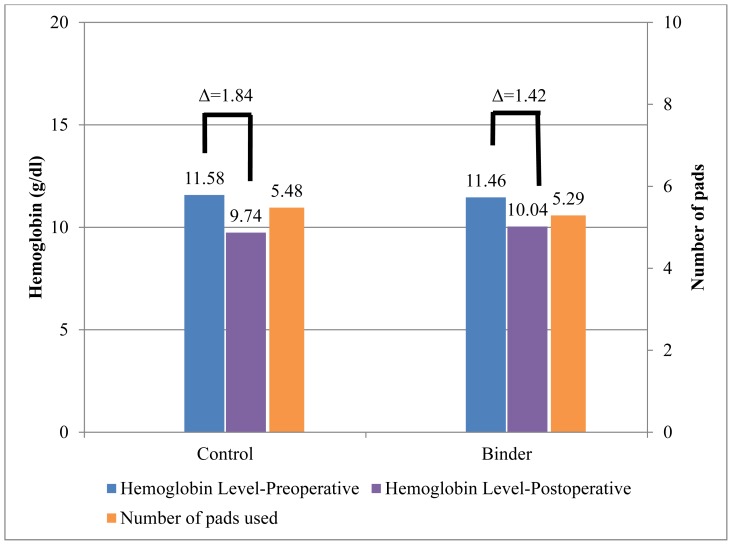
Average hemoglobin levels preoperative compared to postoperative levels, and number of pads used based on randomization group.

There was no difference in pre-operative to post-operative hemoglobin levels by treatment group, but participants in the binder group had a smaller change in hemoglobin levels preoperative to postoperative (p = 0.406; Figure 3). Binder group participants reported using 5.29 ± 2.27 pads compared to 5.48 ± 1.95 pads for the control group participants, but results were not statistically significant (p = 0.381). One patient in the control group received a transfusion.

In regards to adverse events or side effects in each group, two participants receiving the binder indicated that wearing the device for an extended period caused itching. There were no other side effects reported in either group.

## Discussion

In this study, the use of an elastic abdominal binder significantly lowered average postoperative pain scores when compared to the control group. However, differences in hemoglobin concentrations before and after surgery were not statistically significant. There is also no significant difference in the number of pads used between the two groups.

Our results demonstrated that postoperative pain was improved with the abdominal binder. To account for the postoperative pain medication effect, the average dose of pain medication was compared between the intervention and control group. Our findings supported previous findings that an abdominal binder reduces postoperative pain among patients going through major abdominal surgery,^13^ and in regard to postoperative pain, our results were in agreement with the Ghana et al. study.^15^

Karlstrom et al.^9^ reported that 78% of women in their study experienced pain greater than or equal to 4 on the Visual Analog Scale (VAS) during the first 24 hours after cesarean. Since pain may interfere with recovery and impede maternal-infant interactions, results suggested that an abdominal binder may alleviate patient pain during the first 24 hours following surgery. In our study, average pain reported 24 hours postoperatively among the binder group, was similar to the results found by Ghana et al.^15^ In comparison, the patients in the control group in our study had lower average pain compared to their study control group. On postoperative day one, Gillier et al.^14^ reported no difference in VAS scores regarding postoperative pain, but noted a slight difference in scores postoperative day two. However, in both instances, the abdominal binder group reported lower scores for both days, which was supported by our results that postoperative pain is lower among the abdominal binder group.

Our study found that, in general, pain did not interfere with maternal daily functions or activities postoperative regardless of treatment group. However, our study found that with the abdominal binder group, women reported lower pain interference when feeding and bonding with the baby. Although postoperative pain may not prevent a mother from feeding or bonding with the baby, women in greater pain are less likely to breastfeed.^8^,^9^ Even though our study did not look at breastfeeding outcomes explicitly, it demonstrated abdominal binders may reduce pain so mothers can feed and bond with their newborns. Pain interfering with general activity and walking was slightly higher among binder patients, which is in contrast to findings by Cheifetz and colleagues that binders may improve mobility.^13^ However, our patients reported pain interfering with general activity and walking in the 24-hour postoperative time, whereas Cheifetz’s significant findings are reported on postoperative day five. Additionally, our patients may have reported pain interference that may be due to the actual compression and bulkiness of the abdominal binder, not the postoperative pain. Pain interference with postpartum activities should be investigated further, especially if binders improve breastfeeding initiation and mobility.

Change in hemoglobin concentrations and pad counts were not significantly different between the groups, suggesting that the binder did not have a significant effect on 24-hour postoperative blood loss. This was not surprising, since most blood loss in a cesarean is intra-operatively. Ghana et al.^15^ found a statistically significant higher blood loss volume in their control group between baseline and 36 hours. Similarly, based on results from our study, the difference in hemoglobin concentration levels before and 24 hours after surgery were lower among women in the binder group, although not statistically significant. Furthermore, Ghana et al.^15^ used a method presented by Shook et al.^24^ to calculate blood loss based on estimated patient blood volume, and hematocrit levels measured preoperative and postoperative. We are unable to extrapolate on the postoperative blood loss using the pad counts, since estimated blood loss was not determined from the pads; however, we think pad use provides insight regarding blood loss in the postoperative period. Both our study and the Ghana et al. study provided estimates of postoperative blood loss, which should be investigated in future studies. Overall, there may be a possible benefit to binder use and postoperative blood loss at a longer duration postoperative, and this should be investigated further from a large sample size population.

The findings of this trial are generalizable to the population of pregnant women undergoing cesarean delivery; however, caution must be taken when interpreting the effectiveness of the abdominal binder. One limitation of this prospective randomized controlled trial was a smaller sample size and potential reporting bias due to inability to blind patients. Because of the small sample, we were unable to detect statistical significance between the control and intervention group on some important secondary outcomes, such as number of pads used during the first 24 hours postoperatively. Our study did not standardize or validate the level of pad saturation, we simply assumed that women changed their pad as necessary without determining the exact quantity of blood in the pad. Although the same type of pad utilized by our institution was used by all study participants, a future study may consider using a more accurate measure of postoperative blood loss, such as a measuring saturated pads or a menstrual pictogram. Additionally, postoperative hemoglobin concentration may have been reported in the electronic medical record at varying times, so we are unable to report an exact time for the postoperative hemoglobin concentration.

In addition to strengths commonly associated with randomized controlled trials, a strength of this study was limited loss of follow-up. A future study should aim to increase sample size, should consider utilizing validated qualitative measurements and estimations of postoperative blood loss, and determine pain medications used between treatment groups.

In conclusion, this study showed significantly improved lowest-reported pain scores and average pain scores among participants randomized to the treatment group (using the binder). Thus, the use of an abdominal binder may be a cost-effective, non-pharmacologic intervention to reduce postoperative pain after cesarean delivery.

## Figures and Tables

**Table 1:** Patient Demographics and Clinical Characteristics^a^

Characteristic	Binder (n = 29)	Control (n = 27)	*P* value
Age at cesarean (years)	28.5 ± 4.7	27.7 ± 4.4	0.420
Number of previous vaginal births, median (interquartile)	0 (0 – 0)	0 (0 – 0)	0.236
Number of previous cesarean deliveries, median (interquartile)	1 (0 – 2)	1 (1–2)	0.354
Body mass index calculated during 1st visit	28.9 ± 6.6	28.6 ± 6.3	0.913
Gestational age at cesarean (weeks)	39.1 ± 0.3	39.3 ± 0.6	0.094
Infant birth weight (grams)	3632.5 ± 449.2	3525.2 ± 514.8	0.346
Reason for cesarean delivery			1.000
Previous cesarean delivery	26 (89.7%)	24 (88.9%)	
Breech presentation	3 (10.3%)	3 (11.1%)	
Received Epidural	28 (96.6%)	27 (100%)	1.000
Received Duramorph	29 (100%)	27 (100%)	1.000
Average Dose of Pain Medication (in milligrams) within the first 24 hours			
Hydromorphone hydrochloride (IV)	1 ± 0.71	0.92 ± 0.66	1.0000^b^
Morphine (IV)	6.11 ± 2.79	6.77 ± 3.95	0.5418
Nalbuphine hydrochloride	7.5 ± 2.67	11.43 ± 4.76	0.1054^b^
Acetaminophen/hydrocodone	19.71 ± 10.39	17.42 ± 9.8	0.4783
Ibuprofen	888.89 ± 266.67	900 ± 282.84	1.0000^b^
Oxycodone^c^	22.5 ± 24.75	18.33 ± 18.93	
Oxycodone and acetaminophen^d^	11.67 ± 7.64	10 ± 0	
Ketorolac tromethamine	86.54 ± 17.65	86.79 ± 17.01	0.9584
Estimated blood loss (cc)	655.17 ± 183.39	668.52 ± 150.73	0.570
Number of pads used during 24 hours postoperative	5.29 ± 2.27	5.48 ± 1.95	0.381

a All values in Table 1 were presented as mean ± SD, interquartile range, or as the N (%) depending on the characteristics of the variable.

b P-value was calculated based on non-parametric Wilcoxon rank sum test due to small sample size.

c No p-value can be calculated due to only 3 patients in the binder group and 2 patients in the control group received Oxycodone.

d No p-value can be calculated due to only 3 patients in the binder group and 3 patients in the control group received Percocet.

**Table 2:** Postoperative Pain Assessment Questions.

*How Much Has Pain Interfered with Your:*	Binder (n = 29)	Control (n = 27)	Both (n = 56)	*P* value
General Activity (missing=2)	5.1 ± 3.1	4.9 ± 2.4	5.0 ± 2.7	0.767
Mood	2.0 ± 2.5	3.1 ± 2.8	2.5 ± 2.7	0.122
Walking Ability (missing=4)	4.8 ± 3.2	4.6 ± 3.1	4.7 ± 3.1	0.747
Bonding with your Baby (missing=1)	0.7 ± 1.3	1.7 ± 2.8	1.2 ± 2.2	0.295
Feeding your Baby (missing=2)	0.7 ± 1.2	2.0 ± 2.8	1.3 ± 2.2	0.078
Relationships with other People (missing=1)	0.9 ± 1.8	1.0 ± 2.2	1.0 ± 2.0	0.992
Sleep	3.7 ± 3.3	3.7 ± 2.5	3.7 ± 2.9	0.791
Enjoyment of Life	2.1 ± 2.7	1.9 ± 2.5	2.0 ± 2.6	0.959

All values in Table 2 were presented as mean ± SD.

Pain was assessed using a visual analog scale, with 0 representing “Does not interfere” and 10 representing “Completely interferes.”
